# Navigating Sensitive Conversations: Patient Experiences of Sexuality Discussions in IBD Care: A Qualitative Study

**DOI:** 10.3390/nursrep16070219

**Published:** 2026-06-26

**Authors:** Hege Ingrid Sydnes, Marte Langberg Vangen, Kjersti Alsaker, Marit Hegg Reime

**Affiliations:** 1 Department of Gastroenterology, Akershus University Hospital, Sykehusveien 25, N-1478 Lørenskog, Norway; hege.ingrid.sydnes@ahus.no (H.I.S.); marte.langberg.vangen@ahus.no (M.L.V.); 2 Department of Health and Caring Sciences, Faculty of Health and Social Sciences, Western Norway University of Applied Sciences, Inndalsveien 28, N-5020 Bergen, Norway; kjersti.alsaker@hvl.no; 3 National Centre for Emergency Primary Health Care, NORCE Norwegian Research Centre, Årstadveien 17, N-5009 Bergen, Norway; 4 Department of Postgraduate Studies, Lovisenberg Diaconal University College, Lovisenberggata 15B, N-0456 Oslo, Norway

**Keywords:** inflammatory bowel disease, nurse–patient communication, outpatient care, patient experiences, qualitative study, reflexive thematic analysis, sexual well-being

## Abstract

**Background:** Inflammatory bowel disease (IBD) is a chronic condition characterized by persistent inflammation of the gastrointestinal tract. Sexual dysfunction is a common but often overlooked consequence of IBD, affecting approximately half of women and one-third of men living with the disease. Despite the significant role of sexuality in overall quality of life, discussions about sexuality frequently remain absent from clinical encounters between patients and healthcare providers. **Purpose:** This study aims to deepen understanding of how individuals with inflammatory bowel disease experience—and wish to approach—conversations about sexuality with healthcare professionals in specialist clinical settings. **Method:** A descriptive and exploratory qualitative design was employed, using semi-structured interviews with 12 individuals diagnosed with IBD, recruited from two outpatient clinics in Norway. The data were analysed using reflexive thematic analysis. **Results:** Our analysis generated three main themes: (1) sexuality as an overlooked dimension of IBD care, (2) unmet informational needs related to sexuality, and (3) relational prerequisites for discussing sexuality. Sexuality was seldom addressed in participants’ healthcare encounters. Only a minority had been invited into such discussions, and those experiences were typically brief. Some participants preferred not to engage in conversations about sexuality. Reported barriers included awkwardness, embarrassment, stigma, discomfort, and concerns about privacy. Participants also described limited access to reliable information and perceived some healthcare providers as insufficiently knowledgeable or dismissive when the topic was raised. Feeling safe, trusting the provider, and having an established therapeutic relationship were identified as essential conditions for discussing sexuality. **Conclusions:** Sexuality remains largely unaddressed in clinical encounters with individuals living with IBD. The findings reveal a gap between patients’ information needs and the support currently provided. Strengthening healthcare providers’ competence and ensuring access to appropriate resources may help create the trust and safety required for meaningful conversations about sexuality.

## 1. Introduction

Inflammatory bowel disease (IBD), comprising Crohn’s disease and ulcerative colitis, consists of chronic, immune-mediated, relapsing conditions with a rising global burden, affecting an estimated five million individuals worldwide [1]. As no curative treatment currently exists, management focuses on symptom control and the maintenance of remission [2]. Norway is among the countries with the highest incidence rates, with annual incidences of 14.1–16.0 per 100,000 persons for Crohn’s disease and 24.7–28.4 per 100,000 persons for ulcerative colitis [3]. Beyond gastrointestinal manifestations, such as abdominal pain, bloody diarrhoea, faecal urgency, faecal incontinence, and perianal complications, IBD has substantial effects on multiple dimensions of health, including sexual well-being [4,5]. Approximately half of women and one third of men report reduced sexual desire and satisfaction following an IBD diagnosis [6,7,8], indicating that sexuality is a clinically relevant yet often overlooked aspect of living with the disease.

Persons with IBD are at increased risk of sexual dysfunction, including dyspareunia, anorgasmia, erectile dysfunction, and reduced sexual desire, as highlighted by the European Crohn’s and Colitis Organisation (ECCO) [5,9]. Challenges related to sexuality may also have significant relational consequences: approximately 40% of individuals with IBD report that the disease has hindered the initiation of intimate relationships, while 34% state that IBD has contributed to the dissolution of a relationship [10].

Evidence from meta-analyses indicates a significantly higher prevalence of sexual dysfunction among both women and men with IBD compared with healthy controls [7,8]. Identified risk factors include younger age, prior surgery, high disease activity, depressive symptoms, and medication use, particularly corticosteroids [7,8]. Both qualitative studies and cross-sectional cohort studies further contextualize these findings, demonstrating that although sexuality remains an important aspect of life for individuals with IBD, symptoms such as abdominal and pelvic pain, incontinence, fatigue, altered body image, surgical scarring, and perianal disease constitute substantial barriers to sexual activity and intimacy [11,12].

Sexual health is widely recognized as a fundamental component of overall health and well-being. According to the World Health Organization [13], sexual health encompasses the physical, emotional, mental, and social dimensions of well-being related to sexuality, extending beyond the mere absence of disease or dysfunction. Achieving sexual health requires a positive and respectful approach to sexuality, supported by access to high-quality information, awareness of risk factors, appropriate sexual healthcare, and a cultural context that promotes and accepts sexual well-being [13]. Sexuality itself is understood as a central aspect of human life, shaped by biological, psychological, social, cultural, and structural factors, and expressed through a wide range of thoughts, desires, behaviours, identities, and relationships [14]. Together, these perspectives underscore the complexity of sexual health and highlight the need for healthcare systems to address sexuality as an integral part of holistic care.

Given the high prevalence of sexual concerns in this population, structured approaches are needed to support healthcare professionals—particularly nurses, who often have close and sustained patient contact—in addressing sexuality in clinical practice. The PLISSIT model, developed by Annon, offers a pragmatic framework that facilitates the integration of sexual health into routine care [15]. The PLISSIT model enables clinicians to legitimize discussions of sexual concerns (*Permission*), provide basic disease-related information (*Limited Information*), and, when appropriate, offer individualized guidance (*Specific Suggestions*) or refer patients for specialized interventions (*Intensive Therapy*) [15,16]. In addition, the BETTER model represents a complementary communication tool designed to support the initiation and management of sexual health discussions [17]. This model encourages clinicians to *Bring up* the topic, *Explain* that sexuality is a fundamental aspect of life, *Tell* patients that resources are available to address their concerns, consider the appropriate *Timing* of interventions, *Educate* patients about potential sexual side effects of treatment, and *Record* all assessments and interventions in the medical record [17,18]. In Norway, a dedicated national strategy states that health professionals are expected to address sexuality-related issues with patients regardless of age, gender, ethnic background, sexual orientation, or disability [19]. Similarly, ECCO provides guidance on how sexuality should be followed up by health professionals, both in general and specifically for nurses [9,20]. However, neither of these strategies specifies the BETTER or the PLISSIT models as potential frameworks to support conversations about sexuality.

Studies have shown that patients with IBD report that conversations about sexuality with healthcare professionals rarely occur and that healthcare professionals often lack initiative, training, and confidence in discussing sexuality, resulting in unmet needs and negative care experiences [21,22]. Studies also indicate that approximately half of individuals with IBD, regardless of gender, age or disease subtype, do not wish to discuss sexuality with healthcare personnel, even if the topic is raised by staff [23]. Among those who are willing to engage in such conversations, 24% prefer to speak with an IBD nurse, 15% prefer to speak with a physician, while the remainder report no specific preference [23]. Healthcare professionals, for their part, note that patients with IBD seldom initiate questions related to sexuality, and half of the professionals address the topic only occasionally, whereas 23% never discuss it at all [12]. When sexuality is addressed, the discussion most often centres on fertility concerns in women or erectile difficulties in men [12]. Taken together, these findings highlight a persistent gap between the sexual health needs of individuals with IBD and the extent to which sexuality is addressed in clinical practice.

### Aim of This Study

The aim of this study was to explore how individuals living with IBD experience conversations about sexuality with healthcare professionals, with particular attention to their expectations, preferences, and perceived barriers to addressing these topics.

## 2. Materials and Methods

### 2.1. Study Design

A descriptive qualitative design was used to explore participants’ experiences and perceptions. This design is well suited for studies seeking rich, straightforward accounts that remain close to participants’ own words. It allows for the identification of patterns while acknowledging the subjective and individual nature of participants’ experiences. The approach also recognises the researcher’s interpretive role, shaped by prior experience and positionality [24,25]. This study was conducted and reported in accordance with the *Consolidated Criteria for Reporting Qualitative Research* (COREQ) checklist [26], ensuring transparency throughout the research process.

### 2.2. Inclusion Criteria

The inclusion criteria were adults aged ≥18 years with a confirmed IBD diagnosis who were receiving follow-up care in specialist health services, were able to speak and understand Norwegian, and had the capacity to provide and sign informed consent to participate.

### 2.3. Sample and Recruitment

A purposive sampling approach was used to ensure a heterogeneous sample and to increase information power by incorporating variation in participant characteristics [27]. Participants were selected with attention to demographic and clinical factors such as age, gender, duration of disease, IBD subtype, relationship status, and comorbidities. Participants were recruited from two outpatient IBD clinics in Norway, where nurses invited eligible individuals during routine follow-up appointments. Those who expressed interest in this study were either invited to a private room for additional information on days when the researchers were present at the clinic, or they contacted the research team by email to arrange participation. No record was kept of the individuals who were verbally invited by the nurses, and we therefore do not know how many were approached or declined participation. Two individuals initially provided consent but later withdrew after receiving additional information prior to the interview. Neither participant specified a reason for choosing not to take part in this study. None of the researchers were employed at the participating clinics, and they had no prior relationship with or familiarity with the participants. This reduced the likelihood of social desirability bias, as well as other potential sources of bias, including confirmation bias, interviewer bias, role-related power imbalances, and insufficient probing of familiar topics [25,27].

### 2.4. Data Collection

Data were collected between October and December 2024. A semi-structured interview guide was developed in accordance with the guidelines proposed by Kallio et al. [28], informed by a literature review and discussions grounded in clinical experience (Section S1). To enhance the clarity, relevance, and the overall flow of the interview guide—including the introduction of questions and transitions between them—a pilot interview was conducted with a peer supporter and active representative from the Norwegian Intestinal Association, and the guide was further reviewed by an individual living with IBD. Several revisions were made based on their feedback. Some questions were perceived as too extensive and were therefore divided, and two new questions were added about who was present during the conversation and when and where discussions about sexuality would feel most natural. It also became clear that a shared definition of sexuality was needed, as participants might understand the term differently, and this clarification was provided in the information form (Section S2).

Four interviews were conducted via the Zoom digital platform, while eight took place in conjunction with scheduled follow-up appointments at the IBD outpatient clinics, in rooms that ensured a private and conducive interview environment free from distractions. Ten interviews were conducted jointly by two of the researchers (HIS, MLV) to facilitate the observation of nonverbal cues, while two interviews were conducted independently by one researcher (MLV). Before each interview began, the researchers introduced themselves, provided general information about this study, and offered a brief explanation of how sexuality was defined in the context of the research. This approach aimed to establish a relationship characterized by respect and shared understanding [29]. The researchers sought to create a supportive atmosphere, listen actively, and encourage participants to speak freely about a topic that may be considered taboo. Interviews lasted between 10 and 40 min, were audio-recorded with participants’ consent, and subsequently transcribed verbatim and anonymised. Initial transcription was generated using Autotekst [30], after which all recordings were carefully reviewed, and the transcripts were corrected to ensure accuracy and completeness [25].

### 2.5. Data Analysis

Data were analysed using Braun and Clarke’s six-phase reflexive thematic analysis [25,31,32]. In the first phase, the researchers familiarised themselves with the data through repeated reading and listening to the interview recordings. In the second phase, initial codes were generated through line-by-line coding, followed by collaborative discussions to identify meaningful units, which were then organised in a structured coding table. During the third phase, the codes were examined and clustered to identify preliminary subthemes and themes, supported by the creation of a visual mind map. In the fourth phase, the themes and their associated data extracts were reviewed, refined, and reorganised through iterative discussions to ensure analytical coherence and comprehensiveness. In the fifth phase, the final themes were defined and named. In the sixth and final phase, the findings were reported in this article, supported by illustrative participant quotations to contextualise and substantiate the identified themes. Table 1 provides an illustrative example of the steps involved in the analysis process.

### 2.6. Ethical Considerations

Approval was sought from the Regional Committee for Medical and Health Research Ethics (ref. 760140), which determined that this study did not require formal ethical approval. The project was registered with SIKT—the Norwegian Agency for Shared Services in Education and Research (ref. 240926). Approval was also obtained from the clinical managers and data protection officers at both hospitals.

All participants received written and oral information about this study, including its voluntary nature and their right to withdraw at any time without consequences [33]. Written informed consent was obtained prior to data collection. Data were stored securely on the institutional research server in accordance with applicable data protection regulations, with access restricted to authorised members of the research team [30].

## 3. Results

This study is based on individual interviews with 12 individuals living with IBD. Characteristics of the participants are presented in Table 2. Our analysis generated three main themes related to participants’ experiences of discussing sexuality with healthcare professionals: (1) sexuality as an overlooked dimension of IBD care, (2) unmet informational needs related to sexuality, and (3) relational prerequisites for discussing sexuality.

### 3.1. Sexuality as an Overlooked Dimension of IBD Care

Sexuality was rarely addressed in clinical encounters. Approximately half of the participants—across genders—had never discussed sexuality with healthcare professionals. As participant N6 explained: “It has actually never been brought up. Neither when I’ve been on an outpatient check-up with a doctor and nurse, nor when I was hospitalized. No one has asked about sexuality or anything, unless I’ve asked”.

Among those who had discussed sexuality with healthcare professionals, two had done so in contexts unrelated to their IBD. When sexuality was addressed in relation to IBD, the conversations were typically initiated by the participants themselves. Of the three participants who had discussed sexuality in connection with their IBD, two sought information about the impact of IBD on pregnancy and fertility, while one raised concern about how IBD affected sexual functioning. Only one participant reported being asked about sexuality by healthcare professionals. Taken together, these accounts suggest that sexuality is not routinely integrated into IBD care but tend to surface only when patients themselves introduce the topic.

Participants varied in how they interpreted this absence. Some did not perceive the lack of discussion as problematic, whereas others expected healthcare professionals to assume responsibility for introducing the topic—particularly at diagnosis or during periods of disease instability. Participant N8 emphasized: “In these types of situations, the healthcare professionals should initiate that conversation, to begin with. It’s something everyone is wondering about…. I would honestly dare to say that. I think everyone probably has questions about sexuality after this type of diagnosis”.

At the same time, several participants expressed that they did not wish to discuss sexuality within the IBD clinic, either because they felt their sexuality was unaffected or because they preferred to address such matters with their general practitioner, as N11 explained: “Well, here I don’t want it. Because here I don’t need to have that conversation, as I see it. So, no, at the IBD outpatient clinic, where I receive treatment for ulcerative colitis, I do not want to sit and talk about my sex life at the same time. Instead, I would have gone to the GP, probably”.

These differing expectations highlight a potential mismatch between patient needs and clinical practice. They underscore the importance of a proactive yet individualised approach in which healthcare professionals acknowledge sexuality as a legitimate aspect of care while remaining attentive to patient preferences.

Participants identified several barriers that hindered discussions about sexuality, including perceptions of sexuality as taboo, highly private, or culturally sensitive. Feelings of discomfort or embarrassment were common and appeared to limit patient-initiated dialogue, as described by N11: “It’s close and private, so you don’t want to share it with just anyone. The nurse is someone you might see once every eight weeks, so you don’t want to just start talking about your sex life with them. It is perhaps a topic associated with certain taboos and elements of Christian ethics that still linger among us Norwegians”.

Two participants also noted that a large age difference or a difference in gender between patients and healthcare professionals could act as barriers to conversations about sexuality. As participant N6 explained: “There may be an age difference between patient and doctor, in which case it is not so easy to start the conversation … And I think it can be a bit embarrassing to talk to a man, the conversation may be easier with someone of the same gender”.

These barriers may contribute to the continued marginalization of sexuality in IBD consultations. From a practice perspective, participants underscore the need for healthcare professionals to normalize discussions of sexuality as routine components of holistic IBD care, while creating a safe and respectful environment that supports patients’ choices.

### 3.2. Unmet Informational Needs Related to Sexuality

Participants consistently described a lack of information about sexuality in relation to IBD. Information was perceived as particularly important early in the disease trajectory, when diagnosis introduced uncertainty and new concerns. Several participants felt unprepared to understand or manage the potential impact on their sexuality because sexuality was not addressed during initial consultations. As one participant noted, “receiving a diagnosis comes with a whole new set of questions, and sexuality should therefore be acknowledged at the first meeting with a doctor, including clear guidance on whom to contact if questions arise”.

Many participants felt that healthcare professionals underestimated their informational needs. As a result, they often sought information independently—most commonly online—which was described as overwhelming and difficult to navigate. One participant noted that while clinicians may assume certain issues are obvious, newly diagnosed patients “don’t know what to expect,” and that “information is underrated.” These accounts point to a gap in structured, accessible patient education. Participants expressed a need for reliable, diagnosis-specific information on sexuality to be integrated into routine care pathways, particularly at key transition points such as diagnosis or treatment changes.

Participants also described variability in healthcare professionals’ knowledge and engagement with sexuality-related concerns. Superficial or dismissive responses were experienced as invalidating, particularly when sexuality was deprioritised in favour of other clinical issues. One participant described raising sexuality-related questions only to be told that “other things were more important,” which left the participant feeling that their concerns were not taken seriously. Participants emphasized that some clinicians “lack a toolbox in sexuality” and do not approach the patient “as a whole.”

These experiences highlight the importance of professional competence, responsiveness, and a willingness to engage with sexuality as a legitimate dimension of IBD care. When concerns are minimized or overlooked, trust may be undermined and future disclosure discouraged.

### 3.3. Relational Prerequisites for Discussing Sexuality

Trust and a sense of safety were described as essential conditions for discussing sexuality. Participants emphasized the need to be met with openness, respect, and understanding, as well as having sufficient time and a private setting. These relational and contextual conditions were seen as reducing discomfort and enabling honest dialogue about sensitive issues. Participant N3 explained that discussing sexuality with an unfamiliar clinician felt particularly challenging, noting that trust must be built over time: “It can be difficult when you don’t know each other. You’re going to talk about something so vulnerable without knowing how they’ll respond. You need to come regularly and build trust. For me to feel safe, they must meet me with empathy, be nice, understanding, and competent”.

Continuity of care and established relationships played a central role in fostering this sense of safety. Many participants identified the IBD nurse as the preferred professional for conversations about sexuality, citing familiarity, accessibility, and perceived availability. Participant N4 described feeling comfortable raising sensitive issues because of the relationship they had developed: “I have a very good relationship with my IBD nurse. They’re very professional, and I don’t feel there’s anything I can’t bring up”.

Experience and specialist knowledge also contributed to trust. Participants expressed greater confidence in nurses with advanced training or long clinical experience, believing they could provide more secure and informed responses. These accounts suggest that nurses—particularly those in specialized, continuous roles—may be well positioned to initiate and support conversations about sexuality within IBD care. Their ongoing relationships with patients, combined with their accessibility and expertise, create conditions that facilitate sensitive discussions and strengthen patient trust.

## 4. Discussion

This study explored how adult individuals living with inflammatory bowel disease experience conversations about sexuality in specialist care settings. Together, the findings suggest that sexuality is not consistently integrated into routine IBD care, that patients’ informational needs often remain unmet or insufficiently timed, and that relational and organizational conditions—particularly trust, continuity, time, and privacy—shape whether such conversations occur and how helpful they are.

### 4.1. Sexuality as an Overlooked Dimension of IBD Care

The limited attention given to sexuality in routine IBD care has important implications for both clinical practice and patient well-being. When patients are rarely invited to discuss these issues, opportunities for early identification of sexual concerns—and for timely support—are easily missed. The fact that conversations were typically initiated by patients suggests that sexuality remains positioned as an optional or peripheral topic rather than an integrated component of holistic care. Women’s tendency to raise questions about sexuality in relation to pregnancy and contraception reflects broader literature showing that reproductive concerns often initiate sexuality-related dialogue in IBD care [12]. While this aligns with ECCO recommendations for proactive counselling related to pregnancy planning [9], it also highlights a narrow framing of sexuality that risks overlooking other dimensions, such as intimacy, sexual functioning, and relational well-being. When sexuality discussions are limited to reproduction, patients whose concerns fall outside this domain may not receive adequate support.

Reluctance to engage—particularly among some men—echoes earlier reports that a substantial proportion of patients do not wish to discuss sexuality with IBD clinicians [23]. These barriers have important consequences for clinical practice, as they can discourage patients from raising sensitive concerns and contribute to the ongoing marginalisation of sexuality within IBD care. Participants’ descriptions of barriers—such as time pressure, fear of judgment or embarrassment, age and gender differences, limited relational safety, and uncertainty about clinician competence—reflect challenges widely documented in previous research [21,22]. Preferences for same-gender clinicians and discomfort with large age gaps, expressed by several women in this study, further illustrate how interpersonal dynamics shape patients’ willingness to engage in sexuality discussions. Similar patterns have been observed in other healthcare contexts, suggesting that these preferences are not unique to IBD care, but reflect broader norms around trust, vulnerability, and perceived safety in clinical encounters [22,34,35]. In Norway, approximately 89% of nurses are women [36], which may contribute to women feeling more comfortable discussing sexuality with nurses than men do, as some individuals prefer to address sensitive topics such as sexuality with someone of the same gender [37].

Despite its recognised importance, sexuality is often overlooked in consultations between patients with IBD and healthcare professionals [38]. The absence of clear guidelines for identifying and managing sexual concerns in this population further contributes to its marginalisation, and sexuality is rarely incorporated into discussions about treatment goals [39]. The N-ECCO consensus statement underscores the clinical relevance of this issue, noting that concerns related to sexuality may contribute to anxiety and depression, and that nurses should be prepared to identify such problems and refer patients to appropriate specialist services when needed [20]. Normalising sexuality as a routine component of IBD care may help reduce uncertainty about whether such discussions are appropriate. Brief, “permission-giving” statements—consistent with the first step of the PLISSIT model—can lower the threshold for dialogue without presuming that a problem exists [15,16]. For example, clinicians might say: “Many people with this condition have questions about sexuality. Is there anything you would like to talk about?” [16]. Patients have also reported that the absence of direct questions can create doubt about whether sexuality is acceptable to discuss [10,40]. When asking more direct questions, it may be helpful to emphasise that all patients are asked about sexuality regardless of gender, age, or relationship status, and to reassure them that the information shared is treated with the same confidentiality as all other health-related matters [41].

The participants expressed differing preferences regarding discussions of sexuality, yet many emphasised that healthcare professionals should take the initiative. This aligns with previous research showing that patients often want clinicians to open the conversation rather than having to raise the topic themselves [22,42]. At the same time, not all patients are comfortable discussing sexuality, and clinicians must remain attentive to the varied reasons for this reluctance. For some individuals, such conversations may trigger memories of previous negative or traumatic experiences [43], an issue made more salient by the recent rise in self-reported sexual abuse [44]. Against this backdrop, brief, validated screening items may offer a structured way to identify patient needs while maintaining sensitivity [41].

### 4.2. Unmet Informational Needs Related to Sexuality

Participants wanted information about how IBD may impact sexuality, although the timing and format of this information varied. This variability reflects earlier findings that informational needs fluctuate across the disease trajectory [21]. A new chronic diagnosis can be overwhelming and is often accompanied by a high information load, loss of control, and a need to acquire substantial knowledge to manage the condition. These dynamics underscore the importance of prioritising essential content early on and revisiting additional topics, including sexuality, as the disease stabilises [45]. Participants also described difficulty identifying trustworthy and relevant resources—an observation consistent with previous reports [21,22,34]. Although a Norwegian national patient survey ranked sexuality lower among information priorities, newly diagnosed patients must assimilate information across many domains, and lower prioritisation does not imply an absence of need [46]. Notably, Norwegian patient-facing materials on IBD and sexuality remain limited compared with international resources [19,47], potentially contributing to patients’ uncertainty about where to seek reliable guidance. Participants also perceived considerable variation in clinicians’ knowledge and confidence regarding sexuality in the context of IBD care, echoing earlier studies and local audits showing that many healthcare professionals feel underprepared [21,22]. This reflects the limited attention given to sexuality in pre-service education. As a minimum, however, clinicians should be able to recognise their limits and refer patients appropriately, as emphasised in both the PLISSIT model and N-ECCO guidance [15,20,48].

Some participants reported receiving inadequate or dismissive responses when raising sexuality concerns, consistent with previous findings that patients often experience rejection or only brief answers [21,22,42]. Clinicians themselves may face barriers, including uncertainty about their own competence, fear of offending patients, and time constraints [49]. A staged, tailored information strategy may help address these challenges: signalling early that IBD can affect sexuality, offering concise and credible resources, and returning to the topic when patients are ready [45]. Learning and Mastery Centre courses—typically offered soon after diagnosis—provide a practical venue for introducing foundational information. Clinics can also curate a small set of vetted resources to reduce reliance on indiscriminate online searches [19,47]. At the workforce level, integrating sexuality into both pre-service training and continuing professional development is warranted given the persistent competence gaps reported in the literature [21,48].

### 4.3. Relational Prerequisites for Discussing Sexuality

Across genders, participants emphasized that trust, safety, time, privacy, and continuity were essential for meaningful conversations about sexuality. These conditions align with principles of person-centred communication, in which trust is fostered through empathy, attentive listening, and acknowledgement of vulnerability [20,50,51]. As stated in the N-ECCO consensus, nurses are expected to adopt an empathetic, attentive role and provide holistic support [20]. Because conversations about sexuality can be sensitive, careful consideration of when and where they occur is crucial. A stable, open therapeutic relationship can help create the psychological safety needed for such discussions [41].

Participants also highlighted the value of clinicians being transparent about their own limits and outlining clear plans for follow-up or referral. Such honesty can enhance relational safety. Awareness of power asymmetries and mindful positioning are likewise central to ethical, relational care [51]. Trust is further reinforced through competence: patients must feel confident that healthcare professionals possess the necessary knowledge and do not exceed their scope of practice [20].

The IBD nurse emerged as a particularly suitable professional for discussing sexuality, largely due to continuity, accessibility, and the perceived availability of time for personalised follow-up. At the same time, several participants preferred to discuss sexuality with other healthcare professionals, such as their GP. This reflects differing preferences: some patients value a comprehensive, integrated approach within specialist services, while others prefer to keep their IBD care separate from conversations about sexuality. Although the role of IBD nurses is variably defined in Norway, many have substantial experience and postgraduate training, and international organisations have begun to systematise learning opportunities, including e-learning modules on sexuality in IBD [52,53]. Access, however, remains uneven: only 45% of patients across Europe report having access to an IBD nurse, underscoring calls to improve both availability and the quality of specialist consultations [10].

To support meaningful conversations about sexuality, organisations should ensure protected time and privacy and promote continuity of care—ideally through named IBD nurses. Clear local referral pathways, such as those to sexuality services or psychosexual therapy, should be visible to both staff and patients. In settings without IBD nurses, designating a sexual health champion within the team may help coordinate education, triage, and resource provision. Transparent communication about time constraints in brief encounters, combined with scheduled follow-up, can maintain relational safety while acknowledging real-world pressures [51].

### 4.4. Strengths and Limitations

This study employed a descriptive and explorative qualitative design using semi-structured interviews, an approach well suited to exploring experiences and perspectives related to sensitive and under-researched topics [25,27]. Ensuring trustworthiness was a central aim throughout this study. Although qualitative research is sometimes questioned for its scientific contribution, its credibility rests on transparent data generation, rigorous analytic procedures, and reflexive interpretation. To support methodological rigour, we drew on established qualitative quality criteria and attended to reflexivity, credibility, and transferability across all stages of the research process [25,27].

Reflexivity was maintained through continuous consideration of how the researchers’ backgrounds, assumptions, and clinical experience might have influenced data collection and interpretation. Credibility was strengthened through close engagement with the data, iterative coding, and regular team discussions that challenged emerging interpretations and ensured analytic decisions remained grounded in participants’ accounts. Transferability was supported by providing contextual detail about the study setting, participant characteristics, and analytic procedures, enabling readers to judge the applicability of the findings to other contexts [54].

The interviews were conducted by two researchers with clinical experience in IBD healthcare. This background provided familiarity with the clinical context and may have facilitated rapport with participants; however, it also carried the potential risk of influencing both data collection and interpretation. Continuous reflexive attention was therefore required to minimise the influence of assumptions on the analysis [27]. Reflexivity was supported through ongoing team discussions, particularly during the analytic phase where interpretations and decisions were critically examined. Nonetheless, we acknowledge that our perspectives may still have influenced the findings. Such researcher subjectivity is, however, recognised as an inherent and accepted element of qualitative inquiry [25].

Participants were recruited from two smaller IBD outpatient clinics. This sampling strategy provided access to individuals with relevant experiences but may limit transferability to other settings, as all participants were drawn from the same geographical and cultural context. It is also likely that individuals with strong taboos or a history of trauma related to sexuality chose not to participate, meaning that this perspective may be underrepresented and introducing potential bias. Conversely, the inclusion of participants who stated that they did not wish to discuss sexuality, yet still volunteered for this study, may have offered a different and valuable insight into the complexity of patient preferences. As transferability in qualitative research is closely tied to sample characteristics and context [25,27], different perspectives might have emerged in a larger or more diverse sample. For example, the prominent role attributed to IBD nurses in this study may reflect local organizational structures and the availability of specialised nursing roles. Restricting eligibility to Norwegian speakers may have limited the cultural and linguistic diversity of the sample, including perspectives on stigma, privacy, and communication preferences among individuals with IBD who do not speak Norwegian. We also recognize that including English-speaking participants or providing translated materials could have enhanced the representativeness of this study.

Although sufficient time was allocated for each interview, several were relatively short, which may be a limitation and could reduce the depth of exploration, particularly given the sensitivity of the topic, where security, trust, and relationship-building are essential for meaningful dialogue. However, we consider that even the shorter interviews provided valuable and meaningful insights into the topic as we used probing and follow-up questions to support depth within each interview, even when participants chose to give more concise answers.

The interview guide supported consistency, but at times we may have adhered too closely to it, limiting opportunities for follow-up questions and potentially reducing depth [25,29]. At some points, leading questions were introduced to facilitate member checking. This approach may have influenced participants’ responses; however, it also enabled clarification of meaning and supported the validation of the researchers’ interpretations [29].

Most interviews were conducted with two researchers present. While this supported reflexive engagement within the research team, we also recognised that the presence of two interviewers may have influenced the interaction, as some participants appeared slightly less at ease. A single-interviewer format might have facilitated a more relaxed conversational space, although we did not observe that interviews conducted by one interviewer generated noticeably richer or more detailed accounts.

Conducting interviews both via a digital platform (Zoom) and in person may introduce setting-related differences in privacy, comfort, and willingness to disclose sensitive information. In our material, however, we did not observe meaningful differences between the two modes. For some participants, the digital format may even have provided a greater sense of safety when discussing sensitive topics. Rather than aiming for data saturation—a concept not aligned with reflexive thematic analysis—we continued interviewing until we felt we had achieved sufficient depth and richness to meaningfully address the research question. After nine interviews, our emerging interpretations were consistently reinforced across participants. Nevertheless, we conducted three additional interviews because our interviewing skills had become more confident and the later conversations flowed more naturally, providing further nuance and depth to the dataset.

Although this study aimed to explore patients’ experiences of conversations about sexuality with healthcare professionals, most participants had not engaged in such discussions. This limited the amount of data grounded in direct experience. In line with reflexive thematic analysis, we therefore also attended to participants’ wishes, expectations, and imagined preferences, recognising these as meaningful data that illuminate how sexuality is positioned and negotiated within IBD care. Despite this limitation, the interviews generated sufficiently rich and varied accounts to support a nuanced analysis, and we judged the dataset to hold adequate information power for addressing the research question [25,27].

The participants in this study were not asked about disease activity, medication exposure, perianal disease, prior surgery, or stoma status. In retrospect, collecting this information could have provided important contextual detail about participants’ backgrounds and may have enabled a more comprehensive reflection on how different illness trajectories shape experiences and expectations regarding sexuality. Within a reflexive thematic analysis approach, however, our focus was on participants’ meaning-making rather than on mapping themes onto clinical variables, and the absence of these data does not undermine the interpretive aims of this study.

### 4.5. Implications for Nursing Practice

Although this is a small qualitative study, the findings offer important insights into how adults with IBD experience and would prefer to approach conversations about sexuality in clinical care. Understanding patients’ perspectives can support healthcare professionals determine when, how, and by whom sexuality should be addressed during consultations. This study highlights a clear need for simple, validated screening tools that can identify sexuality-related concerns as well as patients’ preferences for discussing them. Current strategies do not specify the BETTER or the PLISSIT models, presenting an opportunity for clinicians to adopt these structured frameworks to guide conversations about sexuality. Such tools could help clinicians initiate discussions without presuming problems, while still respecting patient autonomy. Existing patient-reported outcome measures could also be adapted or supplemented to capture patients’ wishes for dialogue rather than focusing solely on sexual function. Findings from a systematic review suggest that using the BETTER counselling model may improve sexual function, satisfaction, and quality of life while also reducing anxiety and stress. However, the evidence in this review is limited to studies involving women in predominantly Muslim contexts, indicating that the model remains relatively unfamiliar in many Western settings. This highlights the influence of sociocultural factors and underscores the need for culturally sensitive adaptations [55].

Furthermore, the findings indicate a need for clear guidelines and clinical routines that specify how and when information about sexuality should be provided to patients with IBD. A staged approach—informing patients early that IBD may affect sexuality, with opportunities for more in-depth discussion later—may help balance informational needs with the risk of overload. Written materials, including brochures and web-based resources, should be reviewed, updated, and made more accessible, both for patients and as practical support for healthcare professionals.

Adequate education and training are essential for healthcare professionals to address sexuality competently and confidently. Findings from a systematic review indicate that nurses’ ability to provide sexual health education is shaped by several factors, including limited knowledge, beliefs that sexual health is private or a low-priority issue, discomfort discussing the topic, and perceived barriers such as time constraints, unclear responsibility, and insufficient organisational support [56]. Training should therefore include knowledge of sexuality in the context of chronic illness, communication skills for discussing sensitive topics, and opportunities to reflect on personal attitudes and professional boundaries. Integrating sexuality into undergraduate curricula and continuing professional development may help reduce uncertainty and increase clinicians’ willingness to engage in these conversations.

Our study also underscores the importance of relational continuity, time, and trust, particularly highlighting the role of the IBD nurse as a key facilitator of discussions about sexuality. Services should, where possible, ensure continuity of care and allocate sufficient time and privacy for sensitive conversations. In settings with limited access to an IBD nurse, clear referral pathways and designated responsibility within the care team may help ensure that patients’ sexuality-related needs are not overlooked.

### 4.6. Implications for Research

Future research should prioritise the development and validation of brief, patient-centred screening tools, as well as the evaluation of interventions aimed at improving communication, information provision, and professional competence related to sexuality in IBD care.

## 5. Conclusions

This qualitative study provides insight into how patients with inflammatory bowel disease experience—and would prefer to approach—conversations about sexuality in specialist care. Such discussions were largely absent from routine practice and, when they occurred, tended to focus narrowly on fertility. Participants identified several barriers, including limited consultation time, discomfort with the topic, and perceived gaps in professional competence. Participants emphasised the importance of receiving early, accessible, and diagnosis-specific information, supported by relational conditions that foster trust, safety, and continuity of care.

Overall, the findings highlight the need to more systematically incorporate discussions of sexuality into IBD care. Strengthening professional competence, improving access to reliable information, and creating conditions that support open dialogue may help address patients’ unmet needs. Although IBD nurses and physicians were viewed as natural conversation partners in our study, psychologists, pelvic health clinicians, and sexual health services can also play an important role in supporting this patient group, underscoring the value of flexible referral pathways and a holistic, person-centred approach to care.

## Figures and Tables

**Table 1:** Example of the analysis process.

Themes	Preliminary Themes	Codes	Transcript
Sexuality as an overlooked dimension of IBD care	Limited attention to sexuality in IBD care	Had conversation related to IBD (N1, N3, N5, N8)	“I’ve never been asked about it, so I’ve never really thought about it. But it’s not something I can’t talk about either.” (N7)
		Had no conversations related to IBD (N2, N4, N6, N7, N9, N10, N11, N12)	
		Have talked about the topic (N1, N3, N5, N8, N10, N11)	
		Haven’t talked about the topic (N2, N4, N6, N7, N9, N12)	
		Not needed (N1, N2, N3, N4, N5, N6)	
		Not related to IBD (N2, N4)	
		Never received questions (N4, N6, N7, N8, N11)	
		Asked themselves (N1, N6, N8, N10)	
		Received questions (N5)	
		Doesn’t want the conversation (N9)	
		Can be talked about (N4, N7, N8)	
	Divergent preferences for initiating sexuality talk	Healthcare professionals should take the initiative (N1, N2, N3, N5, N6, N7, N8, N10, N12)	“Both can take initiative for the conversation, but I think that healthcare professional should take the initiative first … I’m not the one who should have to take the big step, even if you must take responsibility for yourself, it can feel a bit difficult.” (N3)
		Healthcare professionals must ask (N1, N6, N9, N10)	
		Healthcare professionals must address the topic (N5, N6, N7, N12)	
		Can bring up the topic themselves if needed (N4, N6, N7, N9, N10, N12)	
		Take the initiative themselves (N1, N3, N8)	
		Read the room to understand the need (N4, N5, N7, N8)	
		Questionnaire for reporting needs (N11)	
		Not a conversation you start yourself (N3, N7, N8)	
	Barriers limiting conversations about sexuality	Difficult topic (N1, N2, N3, N4, N6, N7, N10)	“I understand that it can be a little difficult to talk about ulcerative colitis, because it’s about poop and blood and things like that … It becomes extra difficult if you must talk about sex and things like that as well.” (N6)
		Taboo topic (N1, N3, N6, N8, N11)	
		Embarrassing to talk about (N2, N3,N4, N6, N8, N12)	
		Personal/Private theme (N3, N6, N7, N9, N11)	
		Not enough time (N3, N6, N8, N12)	
		Age difference (N3, N6)	
		Gender difference (N1, N6)	

**Table 2:** Demographic characteristics of the study participants (N = 12).

Sample Characteristics	n
Age (years)	
Range: 19–49	
Mean age: 29	12
Duration of IBD (years)	
Range: 5 months–18 years	
Mean duration: 7	12
IBD subtype	
Ulcerative colitis	7
Crohn’s disease	5
Gender	
Male	6
Female	6
Relationship status	
In a stable relationship	5
Single	7
Comorbid conditions affecting sexuality/sexual health	
Yes	3
No	9

## Data Availability

The datasets generated and analysed during the current study are not publicly available due to ethical restrictions protecting participant confidentiality but are available from the corresponding author upon reasonable request.

## Supplementary Materials

The following supporting information can be downloaded at: https://www.mdpi.com/article/10.3390/nursrep16070219/s1. Section S1: Semi-Structured Interview Guide. Section S2. Information Provided to Participants About Sexuality and Sexual Health Prior to the Interview.

## Public Involvement Statement

During the development of the semi-structured interview guide, we conducted a pilot interview with a peer supporter and active representative from the Norwegian Intestinal Association. The guide was also reviewed by an individual living with IBD. Their feedback informed several revisions to improve clarity and relevance.

## Guidelines and Standards Statement

The manuscript was reviewed against the Consolidated Criteria for Reporting Qualitative Research (COREQ) checklist to ensure comprehensive and transparent reporting of the qualitative methods.

## Abbreviations

BETTER model	Bring up, Explain, Tell, Timing, Educate, Record
COREQ	Consolidated criteria for reporting qualitative research
ECCO	European Crohn’s and Colitis Organisation
IBD	Inflammatory bowel disease
N-ECCO	Nurses European Crohn’s and Colitis Organisation
PLISSIT model	Permission, Limited Information, Specific Suggestions, Intensive Therapy
