# Supplementary material for: Navigating Sensitive Conversations: Patient Experiences of Sexuality Discussions in IBD Care: A Qualitative Study
Source: Nurs Rep. 2026 Jun 26;16(7):219. doi: 10.3390/nursrep16070219 (PMC13416404; doi:10.3390/nursrep16070219)
Supplement: Supplementary file 1 [file nursrep-16-00219-s001.zip › nursrep-4338231-supplementary.pdf]

Section S1. Semi-Structured Interview Guide

How do individuals with inflammatory bowel disease experience discussions about sexuality with healthcare professionals?

Background questions

- Can you tell us a little about yourself? Age?
- Do you have Ulcerative Colitis or Crohn's disease?
- How long have you been living with the condition?
- Are you currently in a committed relationship?
- Do you have any other illnesses or disabilities?

Main questions about sexuality

- Have you ever discussed sexuality with a health professional?
- In what situations have you had conversations about sexuality with health professionals?
- If you haven't had such a conversation, what do you feel is missing, and what would make it possible for you to have one?

  Follow-up questions:
  How did you experience the conversation?
  Who initiated the conversation?
  What was the situation like when the topic was brought up? (For example: Were you alone, did you have someone with you, or were several people present?)
  Can you tell us a bit about what can feel difficult in conversations like these?
  Can you elaborate on ...... A little more?

Main questions about wishes around conversations about sexuality

- What are your wishes when it comes to conversations about sexuality with health professionals?

  Follow-up question:
  Who do you think should initiate the conversation?
  When and where do you feel such a conversation would be most comfortable or appropriate?
  Can you elaborate on ...... A little more?

Main questions about barriers in conversations about sexuality

- What do you think may be the challenges or barriers in conversations about sexuality with health professionals?
- What do you think it will take to overcome any challenges or barriers in conversations about sexuality with health professionals?

  Follow-up question:
  Do you personally experience any barriers?
  Can you elaborate on ...... A little more?

Ending

- Is there anything else you would like to mention that we haven’t covered or may have overlooked?

Section S2. Information Provided to Participants About Sexuality and Sexual Health Prior to the Interview

**Definition of sexuality and sexual health**
We want to briefly explain what we mean by sexuality and sexual health so that we share a common understanding.<br><br>The World Health Organization defines sexual health as "*a state of physical, emotional, mental and social well-being in relation to sexuality. It is not merely the absence of disease, dysfunction or infirmity*".<br><br>Sexuality is a fundamental aspect of being human, encompassing sexual identity, intimacy, and reproduction. It is expressed through thoughts, fantasies, desires, behaviours, relationships, and practices.
